# What Is the Likely Impact of Alternative Proteins on Diet Quality, Health, and the Environment in Low- and Middle-Income Countries^☆^

**DOI:** 10.1016/j.cdnut.2023.102064

**Published:** 2024-01-02

**Authors:** Mansha Kapur, Alexis N. Peña, Navya Sreeram, Martin W. Bloem, Adam Drewnowski

**Affiliations:** 1 Department of Biology, Johns Hopkins University, Baltimore, MD, United States; 2 Translational Tissue Engineering Center, Department of Biomedical Engineering, Johns Hopkins School of Medicine, Baltimore, MD, United States; 3 Department of International Health, Bloomberg School of Public Health, Johns Hopkins University, Baltimore, MD, United States; 4 Johns Hopkins Center for a Livable Future, Johns Hopkins University, Baltimore, MD, United States; 5 University of Washington School of Public Health, Seattle, Washington, United States

**Keywords:** alternative proteins, plant-based proteins, environmental metrics, sustainability, protein transition

## Abstract

Alternative protein (AP) foods are proposed to support a global protein transition. Whereas AP food innovation has been a strategy to promote consumption of protein sources with low environmental impact in high-income countries (HICs) diets, their relation to sustainable, high-quality diets in low- and middle-income countries (LMICs) remains to be established. AP foods vary in nutrient profile, processing requirements, costs, and environmental impact. Current literature regarding AP suitability in LMIC contexts is limited. This perspective examined environmental and nutritional metrics that can assess the sustainability of AP in LMICs. Current research areas needed to accurately assess environmental impacts while considering nutritional density were identified. An overview of the usability of relevant AP in both high- and low-resource settings was also explored. Metrics addressing diverse contextual synergies in LMICs, unifying nutritional, environmental, and socioeconomic considerations, were found necessary to guide the integration of AP into LMIC diets.

## Introduction

Contemporary global agrifood production systems are striving to feed a growing population of over 8 billion people. The growing population and fast economic growth in low- and middle-income countries (LMICs) are leading to shifts in dietary preferences. Climate change and other challenges also add pressure to the global food system. The global food system is diverse, with high-income countries (HICs) and LMICs having fundamentally distinct needs. Public health challenges that the food system can address in LMICs include the double burden of malnutrition, noncommunicable diseases (NCD), and fruit and vegetable availability. Drivers of food systems transformation include international policy, cultural shifts, and technological innovation [1]. Sustainable healthy diets are defined by the Food and Agriculture Organization (FAO) and World Health Organization (WHO) as “dietary patterns that promote all dimensions of individuals’ health and wellbeing; have low environmental pressure and impact; are accessible, affordable, safe and equitable; and are culturally acceptable” [2]. Healthy diets and sustainable food systems can help meet most United Nations (UN) Sustainable Development Goals, the Kyoto Protocol, and global average temperature goals and net-zero greenhouse gas emissions (GHGEs) in the Paris Agreement [3]. Shifting consumer demand toward healthy, sustainable diets is one strategy to mitigate climate change and significantly reduce GHGEs [4]. Drivers of dietary shifts in LMICs include rapid changes to the food environment with the addition of modern retailers, globalization, and emerging economies.

Alternative Proteins (AP) are protein-rich ingredients sourced from plants, fungi, algae, insects, or animal cells that are intended to replace conventional livestock products, particularly those with high environmental impact like red and processed meats. AP present the potential to support a transformation of the global food system for equitable access to nutrient-rich and sustainable food. Identifying accurate nutritional and environmental metrics that minimize trade-offs is necessary to assess the role of AP in the global protein transition in HICs and LMICs.

HIC settings have typically taken precedence in established approaches to food system transformation. Based on reference diets with adequate nutrients and within planetary boundaries, sustainable healthy diets are primarily composed of vegetables, fruits, whole grains, legumes (beans, peas, nuts, nut butters, seeds, and soy), and unsaturated oils, with low to moderate portions of seafood and poultry, and/or minimal portions of red and/or processed meat [5]. Many healthy and low environmental impact modeled and/or reference diets do not account for LMIC settings, where the addition of animal-source foods and dairy could address nutrient adequacy challenges. Although it is established that varying combinations of plant- and animal-source proteins are optimal to ensure minimum dietary requirements [6], animal proteins are not affordable. There are limited studies researching how AP products may drive food system transformation in emerging economies. This perspective examines the metrics needed to assess the potential impact of AP on diet quality, human nutrition, and the environment in the context of LMICs.

Broad categories currently used to assess the environmental impact of foods include measurements at the ecosystem level, including land, energy, and water use, and indicators of climate change such as temperature, extreme events, and GHGEs [7]. The customization of scientific models and assessments in local settings, as well as variations that can occur due to differing planetary boundaries and assumptions [8], is critical to accurately assess the role of AP in the global protein transition. AP foods have varying technology requirements, from unprocessed (e.g., lentils) to processed (e.g., cultivated meat). Some processing techniques may improve digestibility or bioavailability of essential amino acids in AP foods.

**FIGURE 1:**
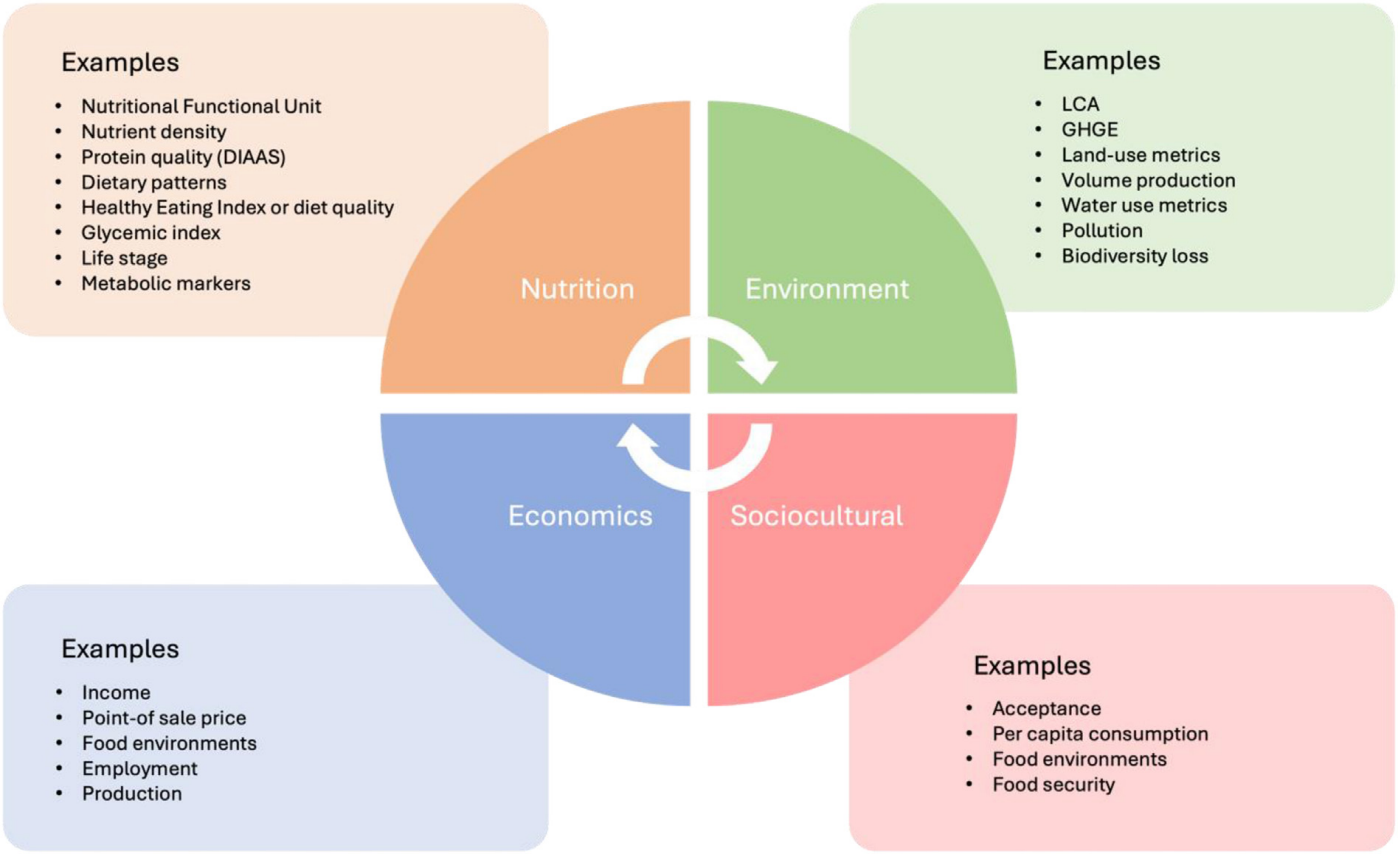
Four dimensions to consider while assessing the impact of alternative proteins in LMICs.

There are several dimensions that influence sustainable healthy diets, including environment, nutrition, sociocultural aspects, and economics. All dimensions need to be considered when balancing benefits and trade-offs (Figure 1). Individual sustainable healthy diets are diverse. HIC and LMIC populations face distinct protein transitions [9]. For a sustainable global food system transformation, the optimization of planetary boundaries is needed to maximize nutritional outputs. Here, we focus on environment and nutrition dimensions as they apply to AP while acknowledging the importance of sociocultural and economic dimensions.

## Current environmental metrics in the context of food production

### Environmental impact assessment tools

Metrics aligned with sustainability goals are particularly crucial within LMIC settings to determine the influence of AP foods on the food value chain. Accurately assessing the environmental impact of food production demands the study of alternative scenarios. Life-cycle assessments (LCAs) serve as comprehensive frameworks for evaluating and managing the environmental footprints of food commodities throughout their lifespan, from production to disposal. This assessment is critical when specialists in food systems, nutrition, or public health evaluate an extensive food supply chain. LCAs measure outcomes from either an attributional or consequential perspective, involving 4 analytical steps: defining objectives and scope, creating a life-cycle inventory, and evaluating the impact and results. The variability in LCA methodologies due to differing boundaries, assumptions, and metrics can complicate interpretation and comparison [10]. Sensitivity analyses are crucial to study the stability of LCA findings against uncertainty factors. Commonly, environmental costs of food production are expressed as GHGEs, land use, and ecosystem services, which include water usage. To quantify environmental impacts, global organizations and initiatives recommend harmonized methodologies to ensure assessments and studies are comparable [11]. For local settings, particular considerations that may alter the net impact of specific indicators include regional disparities, ecosystem variances, or geographical contexts [12].

Land use metrics encompass land occupation and transformation with subcategories such as annual and permanent crops, pasture, intensive and extensive forestry, and urban land. The United Nations Environment Programme (UNEP) Life Cycle Initiative and FAO Livestock Environmental Assessment and Performance (LEAP) partnership recommend using characterization factors as defined by Chaudhary and Brooks for these metrics [13]. Agriculture is the dominant driver in land use, with intensified use potentially leading to ecological damage and soil degradation. Conversely, agroecological strategies that focus on input minimization and biodiversity enhancement can fortify soil health and dietary diversity [14].

The interconnectivity of land, water use, and other ecosystem services is critical, with various methodologies available to assess water use impacts, including water scarcity and withdrawal indicators. Water usage categories cover blue (fresh/surface) [15], green (rainfall and soil moisture), and gray (the volume of freshwater required to dilute pollutants). Food system-related water pollution can cause ecotoxicity from pesticides and eutrophication from fertilizer [16]. Air pollution-induced acidification is another concern.

Environmental metrics, though distinct, are interrelated, with water, energy, and food systems showing dynamic interplays. Defining these nexus synergies necessitates context-specific frameworks, which are currently underdeveloped [17]. Agricultural land use affects water quality, with irrigation and chemical pollution from agricultural inputs impacting it adversely. Land use also influences biodiversity, affecting habitat integrity, species abundance and diversity, and regional/global species richness [18].

#### Synergies

Understanding climate change impacts on food systems involves using standard emission metrics and accounting frameworks, with GHGEs as primary environmental indicators. GHGE sources like deforestation and habitat loss are typically measured per gas in mass units, e.g., atmospheric carbon dioxide equivalents/cap/year. The Intergovernmental Panel on Climate Change (IPCC) does not prescribe specific metrics but recommends using those that convert GHGEs into CO_2_ equivalents [19]. Recognizing total GHGE is important for a comprehensive environmental assessment, given the distinct impacts of different GHGs, including short-lived climate pollutants and their potential for long-term climate effects [20]. Additionally, nitrogen and phosphorous flows are measurable, with atmospheric nitrogen being converted into reactive forms through fertilizer production and legume cultivation. GWP20 and GWP100 values gauge the relative climate impact of gases over 20 or 100 years. Global temperature potential (GTP) quantifies long-term climate effects on global mean temperature, factoring in climate-carbon feedback for all gases [11].

## Nutritional metrics

Nutrient profiling models and sustainable food profiling models are used to model the relationship between nutritional composition and environmental impact [21]. The need for methods that concurrently analyze environmental outcomes and the nutritional quality of food products is pressing [22]. The concept of overall nutrient density of foods was a precursor to nutrient profiling. Methods to capture nutrient density, typically expressed in terms of nutrients per reference amount (100 g, 100 kcal, or serving size), became collectively known as nutrient profiling. Nutrient profiles can be based on nutrients to limit (saturated fat, added sugar, and sodium), nutrients to promote (protein, vitamins, and minerals), or some combination of both [23].

Nutrient Balance Concept (NBC) was developed to assess the overall nutritional quality of meals constituting multiple food items and of total diets [24]. It numerically describes the content of qualifying and disqualifying nutrients in individual food items independent of portion size for simple food components, complex meals, or total diets. It provides an original assessment of overall nutrient quality of meals and diets with numerous food items alongside nutrient complementarity. A mixture of foods from multiple sources yields a higher score as complementarity. Nutrient complementarity and balance are the main innovations in NBC(24). Nutrient-Rich Foods (NRF) index is used to measure nutrient density. The index is a formal scoring system that ranks foods based on their nutrient content. NRF algorithms can be applied to individual foods, meals, menus, or total diet [25].

There are 3 complementary indicators of nutritional quality of a national average diet. Nutrient Balance Score reflects the micronutrient density of individual diets or food items. Disqualifying Nutrient Score indicates whether unhealthy nutrient intake is above the maximum recommended levels. The percentage of population with adequate nutrient intake is a population level indicator estimating the proportion that obtains adequate amounts of essential nutrients [26].

A scoping review evaluated 48 dietary metrics against the 16 guiding principles of sustainable healthy diets and found a lack of adherence, especially concerning sociocultural and environmental factors. Plant-based diets, Mediterranean-style diets, and dietary approaches to stop hypertension (DASH) diets have been found to strongly correlate with decreased environmental impacts compared with current average dietary intakes. However, there is no existing dietary metric that captures all the principles of sustainable healthy diets [27].

### Nutritional functional unit: integration of nutrition and the environment

Nutrition and environmental impacts are assessed in LCA studies, either in parallel or through methods that integrate both. An early example is Barilla’s “Double Food and Environmental Pyramid.” One pyramid is based on the principles of the Mediterranean diet. The environmental pyramid, contrarily, reclassifies food based on the relative magnitude of its environmental impact [28]. The parallel approach reports the environmental impact of a food, meal, or diet obtained from an LCA separately from nutritional value in the impact assessment. There are several advantages to using parallel approaches. They are straightforward, easy to use, and to interpret. The method specifies variability in environmental impact and nutritional value separately where improvements are most required. The current main challenge is that the approach has limited ability to give guidance based on the combined environmental impact and nutritional value. An integrated approach incorporates nutrition-based functional units (nFUs) [16], which introduce some nutritional aspects into the LCA methodology. nFUs may be useful to account for the differences in nutritional content and quality between foods, meals, and diets. Advantages of nFUs include their suitability for use in applications requiring an integrated score that captures both environmental impact and nutritional value. This metric is ideal for relative comparisons of foods. A disadvantage, however, is that they are less transparent. A lack of established threshold values additionally makes it difficult to compare absolute values. Therefore, further research and common agreements among the scientific community are needed. Clear reporting if the approach is solely nutrient based or includes nutritional aspects (bioavailability, digestibility, product matrix, or meal effects) is recommended [17]. Another best practice recommendation is to consider the nonessential nutrient components of food items wherever possible, in addition to their impact on the bioavailability of nutrients. Examples of non-nutrient components of food items that can positively impact humans include dietary fibers and phytochemicals [16]. Wherever possible, the potential losses in nutrients and nutritional quality during food processing and preparation need to be considered(16). While using a nutrient index or nutrient-density score, the nutrient content in food items in relation to the recommended or reference daily intake values of the target populations can be useful. The requirements for nutrients vary by age, sex, life stage, and physical activity, which is why the most optimal reference intake in an nLCA would be a reference developed for the specific target group that would consume the foods assessed [16].

The functional aspects of several foods are difficult to define; they can provide pleasure and satiety in addition to nutrients. Food is also involved in social and cultural exchange. A 2021 FAO report was unable to choose among the multiplicity of possible nFUs arising from the inclusion of all functions, warranting the claim that a comprehensive inclusion is often not possible [16].

The functional unit (FU) needs to be carefully chosen to be compatible with the goal and scope of a food LCA study. The factors that need to be considered are reasons for which the study is being conducted, intended application, and audience. nFU is often useful, but nutrients are not required to be a part of the FU in a Nutritional Life Cycle Assessment (nLCA) study. This is because different functions of foods can be taken into consideration in an LCA study. To progress toward a harmonized environmental and nutritional LCA methodology, there are still several research priorities to be considered. In impact assessments, research on the potential harmful effects on human health due to consumption of food items takes place at an early stage. There are other categories to consider in an nLCA of food items, which include climate change, water use, land use, eutrophication, ecotoxicity, and other human health impacts [16].

## HICs and LMICs: Distinct Protein Transitions

In growing LMIC economies, the intake of more expensive protein sources such as meat is increasing, resulting in improved access to adequate nutrition. In observation of Bennett’s Law, LMIC populations are replacing purchases of affordable carbohydrates with minimal nutrient density with nutrient-dense protein sources [9]. Protein-rich foods, such as animal source foods (ASF), are seen as a status symbol of wealth and may be influencing the shift in dietary preferences as individuals aspire toward a higher social status, as observed in HICs [29]. Thus, AP face dual challenges in LMICs: an increasing demand for nutrient-dense protein sources and the double burden of malnutrition [30]. Malnutrition has been attributed to a lack of protein over a general lack of calories [31]. Addressing the demand positions LMICs at risk of emulating the environmental detriment of contemporary HIC consumption patterns. AP must offer comparable protein quality and nutrient density to be considered viable alternatives to animal-source proteins and ensure a transition toward sustainable, nutritious, and equitable food systems in LMICs.

The need to optimize both nutritional and environmental aspects of food production suggests trade-offs can exist. A 2021 case study in Indonesia observed this compromise. The most nutritional dietary scenario did not have the lowest environmental impacts, and vice-versa [15]. A reliable assessment of AP in dietary recommendations is, thus, critical to food policy. Current recommendations by the FAO do not acknowledge the roles of AP products in LMIC food systems [32]. Similarly, the EAT-Lancet reference diet [5] does not consider low-economic and natural resource availability cases and LMIC contexts. Quantification of the global median cost of the “most affordable” EAT-Lancet diet based on 2011 retail prices across 159 countries revealed that it exceeded the financial capacity of over 1.58 billion people [33]. Additional reviews emphasize similar omissions of socioeconomic determinants in sustainable diets. Improving and integrating available environmental, socioeconomic, health, and nutritional metrics, addressing both LMIC and HIC contexts, can effectively guide AP assessment within international recommendations.

## Conclusion

Various environmental and nutritional indicators were developed with precedence for HIC settings. The reliability of environmental metrics varies across different contexts, and the types used in an LCA can determine its completeness. The selection of metrics can also be an a priori decision, potentially introducing bias [10]. Global and regional frameworks for assessments may not be relevant or accurate for local settings or may not apply to specific subpopulations. Models and frameworks should also use socioeconomic indicators and, more broadly, human health indicators that help identify health risk outcomes. Further development of scientific frameworks to quantitatively assess water-energy-food synergies is needed. Nutritional metrics must be adaptable to country-specific nutritional makeups; environmental metrics must be considered alongside nutritional and socioeconomic metrics to assess the capacity of AP to solve both nutritional and environmental challenges.

Unifying accurate environmental and nutritional metrics can inform the integration of AP within LMICs and HICs. Assessments of AP impacts must reflect variances in cultural, economic, and environmental factors regarding a country’s capacity for food systems reform and address distinct transitions in protein sourcing and demand. Optimal environmental and nutritional considerations are contingent on these metrics accounting for country-specific contexts and, thus, facilitating informed approaches to the introduction of AP as viable agents of a healthy and sustainable future. Within emerging economies, there is an opportunity to learn from historic outcomes in HICs and to identify distinct needs in those countries when deciding on shifts in food production practices, facilities to develop, and agricultural and technological adaptations to improve nutrition while staying within planetary boundaries.

### Author contributions

The authors’ responsibilities were as follows—MK, ANP, and NS wrote the manuscript. AD and MB provided critical review and helpful suggestions. All authors read and approved the final manuscript.

### Funding

The *Sight and Life* Foundation provided funding to support the open access charges for this special supplement.

### Conflict of interest

MK, ANP, and NS are voluntary members of a chapter of the Alternative Protein Project at Johns Hopkins University that collaborates with the Good Food Institute and received no funding. AD is a member of the Nestlé Scientific Advisory Board, chairs the Expert Panel of the Friesland Campina Institute, and is an invited member of the Carbohydrate Quality Expert Panel supported by Potatoes USA. AD has received grants, contracts, and honoraria from entities, both public and private, with an interest in dietary nutrient density and nutrient profiling of foods. MB has no disclosures to report.
